# Evaluation of management of desmoid tumours associated with familial adenomatous polyposis in Dutch patients

**DOI:** 10.1038/sj.bjc.6605997

**Published:** 2010-11-09

**Authors:** M H Nieuwenhuis, E M Mathus-Vliegen, C G Baeten, F M Nagengast, J van der Bijl, A D van Dalsen, J H Kleibeuker, E Dekker, A M Langers, J Vecht, F T Peters, R van Dam, W G van Gemert, W N Stuifbergen, W R Schouten, H Gelderblom, H F A Vasen

**Affiliations:** 1 The Netherlands Foundation for the Detection of Hereditary Tumours, Rijnsburgerweg 10, Poortgebouw Zuid, 2333 AA Leiden, The Netherlands; 2 Department of Gastroenterology and Hepatology, Academic Medical Centre, Amsterdam, Meibergdreef 9, 1105 AZ Amsterdam, The Netherlands; 3 Department of Surgery, Maastricht University Medical Centre, P. Debyelaan 25, 6229 HX Maastricht, The Netherlands; 4 Department of Gastroenterology, Radboud University Nijmegen Medical Centre, Geert Grooteplein Zuid 10, 6525 GA Nijmegen, The Netherlands; 5 Department of Surgery, Atrium Medical Centre Heerlen, Henri Dunantstraat 5, 6419 PC Heerlen, The Netherlands; 6 Department of Surgery, Isala Clinics Zwolle, Dokter van Heesweg 2, 8025 AB Zwolle, The Netherlands; 7 Department of Gastroenterology and Hepatology, University Medical Centre Groningen, University of Groningen, Hanzeplein 1, 9713 GZ Groningen, The Netherlands; 8 Department of Gastroenterology and Hepatology, Leiden University Medical Centre, Albinusdreef 2, 2333 ZA Leiden, The Netherlands; 9 Department of Gastroenterology and Hepatology, Isala Clinics Zwolle, Dokter van Heesweg 2, 8025 AB Zwolle, The Netherlands; 10 Department of Gastroenterology and Hepatology, Elisabeth Hospital Tilburg, Hilvarenbeekseweg 60, 5022 GC Tilburg, The Netherlands; 11 Department of Surgery, Erasmus University Medical Centre Rotterdam, Postbus 2040, 3000 CA Rotterdam, The Netherlands; 12 Department of Clinical Oncology, Leiden University Medical Centre, Albinusdreef 2, 2333 ZA Leiden, The Netherlands

**Keywords:** desmoid tumour, desmoid-type fibromatosis, familial adenomatous polyposis, management

## Abstract

**Background:**

The optimal treatment of desmoid tumours is controversial. We evaluated desmoid management in Dutch familial adenomatous polyposis (FAP) patients.

**Methods:**

Seventy-eight FAP patients with desmoids were identified from the Dutch Polyposis Registry. Data on desmoid morphology, management, and outcome were analysed retrospectively. Progression-free survival (PFS) rates and final outcome were compared for surgical *vs* non-surgical treatment, for intra-abdominal and extra-abdominal desmoids separately. Also, pharmacological treatment was evaluated for all desmoids.

**Results:**

Median follow-up was 8 years. For intra-abdominal desmoids (*n*=62), PFS rates at 10 years of follow-up were comparable after surgical and non-surgical treatment (33% and 49%, respectively, *P*=0.163). None of these desmoids could be removed entirely. Eventually, one fifth died from desmoid disease. Most extra-abdominal and abdominal wall desmoids were treated surgically with a PFS rate of 63% and no deaths from desmoid disease. Comparison between NSAID and anti-estrogen treatment showed comparable outcomes. Four of the 10 patients who received chemotherapy had stabilisation of tumour growth, all after doxorubicin combination therapy.

**Conclusion:**

For intra-abdominal desmoids, a conservative approach and surgery showed comparable outcomes. For extra-abdominal and abdominal wall desmoids, surgery seemed appropriate. Different pharmacological therapies showed comparable outcomes. If chemotherapy was given for progressively growing intra-abdominal desmoids, most favourable outcomes occurred after combinations including doxorubicin.

Familial adenomatous polyposis (FAP) is a dominantly inherited cancer predisposition syndrome, caused by mutations in the adenomatous polyposis coli (*APC*) gene. Carriers of the mutated *APC* gene develop hundreds to thousands of adenomatous polyps in the colon and rectum, leading to a nearly 100% cancer risk by the age of 40 years (Lynch *et al*, 2008). By performing a prophylactic colectomy, the risk of death due to colorectal cancer is decreased. Among FAP patients, a spectrum of extra-colonic manifestations is often observed, including duodenal cancer and desmoid tumours. These manifestations are currently the most common causes of death after colorectal cancer (Arvanitis *et al*, 1990).

Desmoid tumours or aggressive fibromatoses are histologically benign proliferations of fibro-aponeurotic tissue (Goldblum and Fletcher, 2002). In the general population, the incidence of desmoids is about 3 per million per year, and the tumours are mainly located in the extremities or in the abdominal wall (Fallen *et al*, 2006). Of all patients presenting with a desmoid tumour, at least 7.5% has FAP or will develop FAP later in life (Nieuwenhuis *et al*, 2010, submitted for publication). In the total FAP population, desmoid tumours develop in about 10–30% and are usually located in the mesentery of the small bowel (Fallen *et al*, 2006). Desmoids range from small, indolent, or even regressive tumours to large and progressive growing neoplasms causing obstruction of vital organs. Desmoid tumours do not metastasise, although they can present as multifocal disease.

Treatment of FAP-related desmoid tumours is controversial (Sleijfer, 2009). As desmoid tumours are rare, and show a variable disease course, the effectiveness of treatment is difficult to determine. There are no randomised controlled trials. Usually, extra-abdominal and abdominal wall desmoid tumours are removed surgically, but two recently published reports argued a wait-and-see policy for patients in which surgery would result in major functional or cosmetic defects (Bonvalot *et al*, 2008; Stoeckle *et al*, 2009). For intra-abdominal desmoid tumours, surgery is not recommended because surgical resection is complicated or impossible in most cases, and because of high recurrence rates (Rodriguez-Bigas *et al*, 1994). Furthermore, there is evidence that tissue damage is a risk factor for desmoid development (Clark *et al*, 1999; Bertario *et al*, 2001). The most frequently used pharmacological therapies include non-steroidal anti-inflammatory drugs (NSAIDs), hormonal agents, biological agents, and cytotoxic chemotherapy (Janinis *et al*, 2003; Tolan *et al*, 2007). Currently, most guidelines recommend a stepwise approach, starting with NSAIDs (preferably sulindac). If this is not effective, hormonal therapy is added, most commonly consisting of tamoxifen or toremifene. Fast growing desmoid tumours not responsive to these agents are treated by cytotoxic chemotherapy or surgery (http://www.nccn.org; Janinis *et al*, 2003; Latchford *et al*, 2006; Melis *et al*, 2008; Casali and Blay, 2010).

In the present study, we retrospectively evaluated long-term outcome of Dutch FAP patients with desmoid tumours, undergoing surgical, and pharmacological therapies. First, we assessed the effectiveness of surgical *vs* non-surgical strategies for intra- and extra-abdominal desmoid tumours, and second, we assessed the effectiveness of various pharmacological modalities in desmoid tumour treatment.

## Materials and methods

### Patients

The FAP database of the Netherlands Foundation for the Detection of Hereditary Tumours was used for the study. This national database comprises medical data on over a thousand FAP patients. Patients gave written consent to register their personal and medical information. A total of 78 patients with desmoid tumours were identified. Patient characteristics, genetic data, and medical information were retrieved from the database. The study was approved by the Medical Ethics Committee.

Desmoid localisation was defined as ‘at least intra-abdominal’ or ‘extra-abdominal and in the abdominal wall’. For all patients, the type of primary therapy for the first desmoid tumour was determined. If surgery was performed due to the severity of desmoid symptoms or with the aim of removing the desmoid tumour, patients were categorised into the ‘surgery’ group. All patients who received conservative treatment (wait-and-see or medication), and patients whose desmoid tumour was detected coincidentally during another surgical procedure, without resection, were categorised into the ‘non-surgery’ group.

Time from diagnosis of the desmoid tumour to progression of desmoid tumour growth was calculated. Progression of desmoid tumour growth was defined as tumour growth causing clinical symptoms. Also, for each patient, the status of desmoid growth at the end of follow-up was assessed and categorised into either ‘regression or stabilisation of tumour growth’ or ‘progression of tumour growth’.

### Data analysis

Baseline characteristics between the groups were analysed by univariate analysis (Student's *t*-test for numerical variables, *χ*^2^-test for categorical variables). Progression-free survival (PFS) was calculated by the Kaplan–Meier method. Univariate analysis was performed using the log-rank test. Statistical analyses were performed using the Statistical Package for Social Sciences (SPSS) version 16.0 (Chicago, IL, USA).

## Results

### Group description

Between January 1978 and January 2010, 78 FAP (34 males) patients had developed at least one desmoid tumour. Desmoid localisations were as follows: 49 (62.8%) intra-abdominal, 13 (16.7%) involving both the mesentery and the abdominal wall, 13 (16.7%) abdominal wall only, 2 (2.6%) trunk, and 1 (1.3%) head/neck. The median size of the desmoids was 7 cm, ranging from 1 to 24 cm. Fifty-six patients were treated at a tertiary referral centre, 22 in a local hospital. The median follow-up period from diagnosis of desmoid to the last observation was 8 years, ranging from 0 to 29 years (Table 1).

### Surgery *vs* non-surgical management for intra-abdominal desmoid tumours

**Figure 1:**
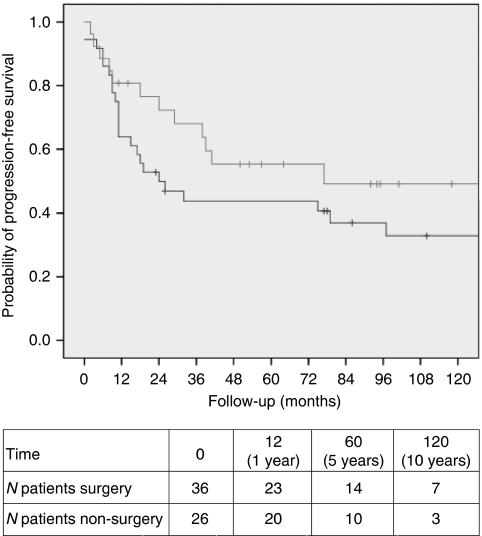
Progression-free interval after primary surgical (black line) and non-surgical (grey line) treatment for mesenterial desmoid tumours in FAP patients (log-rank test, *P*=0.163).

The group with ‘at least intra-abdominal’ desmoid tumours consisted of 62 patients. In 36 patients, primary treatment consisted of surgery with the intention to remove the intra-abdominal desmoid tumour. Twelve of these patients received desmoid-targeted medication immediately after surgery. Primary treatment was non-surgical in 26 patients (17 wait-and-see policy and 9 medication). The surgery and non-surgery groups were comparable for sex, age at first desmoid, size of first desmoid tumour, and duration of follow-up (Table 1). None of the intra-abdominal desmoid tumours could be resected entirely. The probability of PFS for the surgery group was 63.9%, 43.8%, and 32.9% after 1, 5 and 10 years, respectively. In the non-surgical group, these percentages were 80.8%, 55.3%, and 49.1%, respectively (log-rank, *P*=0.163) (Figure 1).

When considering desmoid status at the last observation, the majority of desmoid tumours had become stable or regressive in both the surgery and non-surgery groups (69% and 77%, respectively, *P*=0.515). In the surgery and non-surgery groups, 25% and 19%, respectively, died from desmoid disease (*P*=0.783) (Table 1).

### Extra-abdominal and abdominal wall desmoid tumours

**Figure 2:**
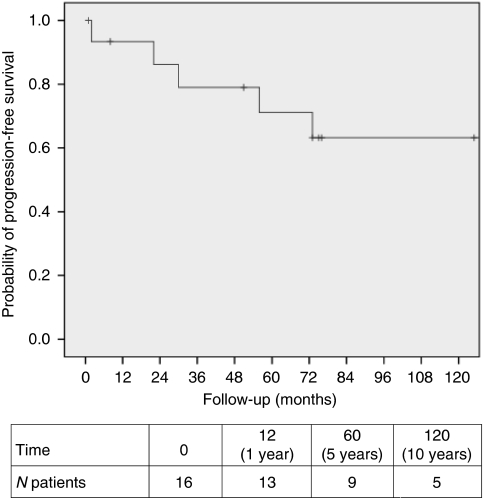
Progression-free interval after primary treatment for extra-abdominal and abdominal wall desmoid tumours in FAP patients.

Sixteen patients had extra-abdominal desmoid tumours (Table 2). Thirteen patients had a desmoid tumour in the abdominal wall (3 male patients and 10 female patients), two male patients had desmoids at the thoracic wall and back, and one female patient had a desmoid tumour localised in the muscles of the neck. Fourteen (87.5%) of the tumours were treated surgically, with seven R_1/2_ (microscopically/macroscopically irradical), six R_x_ (unknown surgical margin), and one R_0_ (radical) excision. One, 5, and 10 years after primary surgery, 93.3%, 71.1%, and 63.2% of patients were free of progression. In most patients, progression was observed within 6 years after primary surgery (Figure 2). None of the patients died from desmoid disease. When considering desmoid status at the last observation, three quarter of the desmoid tumours had stabilised or regressed.

### Effectiveness of pharmacological treatment

**Figure 3:**
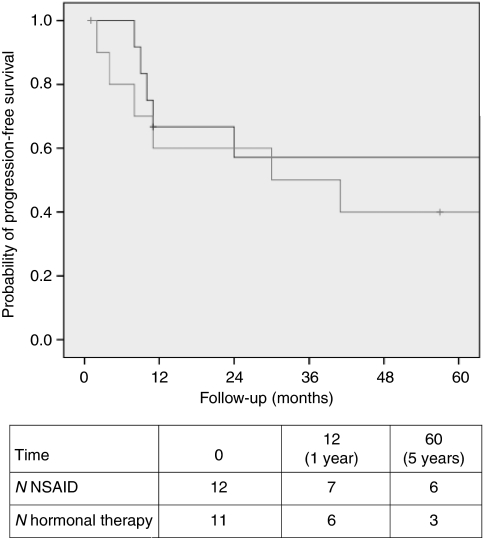
Progression-free interval after NSAIDs (*n*=12, black line), and hormonal therapy or combination therapy (*n*=11, grey line), irrespective of previous surgery (log-rank test, *P*=0.111).

Various pharmacological agents were used, including NSAIDs (sulindac and celecoxib) and hormonal medications (tamoxifen, toremifene, LHRH-agonists, and anastrozole). For all patients who received medical treatment as primary therapy, irrespective of previous surgery, survival rates were calculated. After 5 years of follow-up, the PFS rates were similar after treatment with NSAIDs and hormonal medications including combination therapy, as shown in Figure 3 (log-rank, *P*=0.111). A small subset of patients had received other drugs, including prednisone, interferon, and colchicines. After these medications, both positive as well as negative effects on desmoid tumour growth were reported, but patient numbers were too small to perform statistical analysis.

### Cytotoxic chemotherapy and imatinib

Ten patients received cytotoxic chemotherapy, and three patients had treatment with imatinib. Effects and complications of these therapies are summarised in Table 3. The most frequently used chemotherapy was doxorubicin, in combination with other agents such as DTIC, carboplatin, and ifosfamide. Effects of chemotherapy were variable. Four patients eventually had regression or stabilisation of tumour growth, and five patients had progression of tumour growth. One patient died only a few days after the first session of chemotherapy due to a massive pulmonary embolism, caused by pressure of the desmoid tumour on the large veins. Another patient bled to death due to fistulas and abscesses after chemotherapy. Furthermore, three patients developed severe complications as fistulas and abscesses (after 5, 18, and 60 months, respectively) besides the known spectrum of side effects associated with cytotoxic chemotherapy.

In the three patients receiving imatinib (of which two also had received chemotherapy), variable outcomes were seen, but the follow-up intervals were limited.

### Radiotherapy and embolisation

A total of five patients were treated by radiation therapy. Three patients had radiation therapy for intra-abdominal desmoids. In one of them, the tumour size decreased, enabling surgery. The two other patients had stable desmoids during 6 years and progression within 1 year, respectively. Two patients had radiation therapy for extra-abdominal desmoid tumours: a patient with trunk desmoids had progression within a few months, and another patient had regression of an abdominal wall desmoid after irradiation; however, this patient developed serious radiation enteritis.

In two patients, the desmoid tumours were treated by embolisation. However, in both patients this treatment failed as the desmoids did not have large supplying vessels.

## Discussion

The present study demonstrates that for intra-abdominal desmoid tumours, similar PFS rates were observed after surgical treatment and a more conservative approach. None of the intra-abdominal desmoids could radically be resected by surgery, but at the end of the follow-up period, two thirds of the intra-abdominal desmoids showed regression or stabilisation of tumour growth. About one fifth of the patients died due to complications of an intra-abdominal desmoid tumour. Most patients with abdominal wall and extra-abdominal desmoid tumours were treated surgically. The PFS rates were greater than after surgery of intra-abdominal desmoids, and at the end of the follow-up period, in 75% of the patients the tumours had stabilised or decreased in growth. None of these patients died from desmoid disease. Evaluation of pharmacological agents showed comparable PFS probabilities after NSAIDs and hormonal agents including combination of both medicines. Effects of chemotherapy were variable, with doxorubicin-based regimens being most effective.

The optimal treatment of intra-abdominal desmoids is unknown. Previous studies reported high recurrence rates after surgery and a low success rate of radical removal of desmoid tumours (Rodriguez-Bigas *et al*, 1994; Heiskanen and Järvinen, 1996). On the other hand, favourable outcomes have been reported after surgery performed by experienced surgeons in carefully selected patients (Latchford *et al*, 2006). In our series, none of the desmoids could be radically resected and the PFS was similar after surgery compared to conservative treatment. Based on these findings, a conservative approach appears to be the preferred choice in patients with large stable or slowly growing desmoids. Only in cases of progressively growing desmoids, with complications such as obstruction of the small bowel, surgical treatment might be an option. In such patients, minimal surgery (intestinal bypass) could be performed. In patients with obstruction of the ureter, stenting of the ureter might be indicated. These conclusions support the current guidelines on the treatment of desmoid tumours (http://www.nccn.org) (Casali and Blay, 2010).

Extra-abdominal and abdominal wall desmoid tumours are generally more suitable for surgical therapy than mesentery desmoids. Previous studies reported mainly good outcomes after surgery, although recurrence was common after excision (Clark *et al*, 1999; Melis *et al*, 2008). Recent reports proposed a wait-and-see policy for patients in which major surgical defects are expected, because spontaneous regression or tumour stabilisation is not uncommon (Bonvalot *et al*, 2008; Fiore *et al*, 2009; Stoeckle *et al*, 2009). In our series, the majority of extra-abdominal and abdominal wall desmoids was resected, with overall favourable outcomes, despite only few radical resections. Based on this and previous studies, surgery seems to be safe for extra-abdominal and abdominal wall desmoid tumours. In patients in which surgery would result in serious defects, a wait-and-see strategy should be considered.

Commonly used pharmacological agents are NSAIDs and hormonal agents. One systematic review showed favourable outcomes after using NSAIDs and hormonal agents, although the results might be confounded by successful case reports (Janinis *et al*, 2003). Another prospective study showed the effectiveness of high-dose tamoxifen (120 mg) and sulindac (300 mg) in 9 out of 13 patients (69%), compared to stabilisation after surgery and medication in only 1 out of 4 patients (25%) after 10 years of follow-up (Hansmann *et al*, 2004). Based on these results, the authors advised high-dose tamoxifen and sulindac as the primary treatment for FAP-related desmoid tumours. Recently, another retrospective study reported effective hormonal therapy for desmoid tumours (De Camargo *et al*, 2010). Our study showed no significant differences in PFS rates between NSAIDs and hormonal treatment including a combination of both medicines. The PFS was about 50% at 5 years of follow-up. However, patients in our study received various doses of hormonal agents. Possibly, hormonal treatment at higher doses would have led to significant better outcomes. Based on personal experience from our authors (H.G.), the optimal dose of tamoxifen is 40 mg 4 times a day, and for toremifene 60 mg 4 times a day. Based on this and previous studies, treatment with NSAIDs and/or hormonal agents seems to be the best option for large and/or progressive desmoids.

Recently, several studies reported successful treatment of desmoids with pegylated liposomal doxorubicin, with acceptable side effects (Gega *et al*, 2006; Bertagnolli *et al*, 2008; Constantinidou *et al*, 2009; De Camargo *et al*, 2010). All four patients in our study who reached stabilisation or regression after chemotherapy were treated with doxorubicin. Our findings and those of others suggest that (pegylated liposomal) doxorubicin-based chemotherapeutic regimens are effective for patients with progressive, symptomatic desmoid tumours. Inspite of previous promising reports (Heinrich *et al*, 2006; Wcisio *et al*, 2007), imatinib treatment had no evident positive effects in our patients. Long-term effects of targeted therapies are yet to be evaluated. Recently, a study to evaluate imatinib in desmoid tumours was initiated (NCT01137916).

In the past, radiotherapy alone or in combination with surgery was shown to be effective in sporadic, mainly extra-abdominal desmoid tumours (Ballo *et al*, 1999; Nuyttens *et al*, 2000; Lev *et al*, 2007; Guadagnolo *et al*, 2008). Radiotherapy enabled surgery in one of our patients, but in other patients disease progression after radiotherapy was observed. Recently, an EORTC study (EORTC-62991, EORTC-22998, and NCT00030680) was performed to evaluate moderate dose radiotherapy for inoperable desmoid tumours. Results of this study are not yet available. According to the American National Comprehensive Cancer Network guidelines, radiotherapy should be considered only in desmoid tumours located at the extremities (http://www.nccn.org). Embolisation showed not to be a reliable option for desmoid treatment.

In the current study, we evaluated the effectiveness of long-term treatment of desmoid tumours in FAP patients. Nationwide data, both from university hospitals as well as local hospitals were included, thus avoiding potential confounding by patient selection. However, because of the retrospective study design, we were not able to gain information about the selection of patients for certain treatment modalities. Nevertheless, this is a complete and informative series on desmoid treatment to date. For future studies, a prospective, randomised study design would be a more robust approach to this research question.

For clinical practice, we recommend surgery for patients with extra-abdominal and abdominal wall desmoid tumours, unless major surgical defects are expected. For patients with stable intra-abdominal desmoid tumours, both a wait-and-see strategy as well as pharmacological treatment are appropriate. Cytotoxic chemotherapy may be effective in patients with progressively growing desmoids. In the case of severe complications, including ileus, perforations, and abscesses, surgery is indicated.

In conclusion, desmoid disease is a heterogeneous disease entity, with various treatment modalities. Clustering of desmoid patients in some specialised referral centres will benefit treatment and follow-up, and enables further research into this controversial topic.

## Figures and Tables

**Table 1:** Characteristics and follow-up data of FAP-related mesenterial desmoid tumours, according to primary surgical treatment *vs* non-surgical treatment

	**Primary treatment**		
	**Surgery (*N*=36)**	**No surgery (*N*=26)**	***P*-value**
*Sex, male*			
*N* (%)	16 (44)	13 (50)	0.665
			
*Age at first DT (years)*			
Median, min–max	30, 15–54	35, 14–51	0.396
			
*Size first DT (cm)*			
Median, min–max	9.5, 1–20	6.5, 2–24	0.568
			
*DT progression, N* (%)	26 (72)	13 (50)	0.074
			
*Time to first DT progression (months)*			
Median, min–max	13, 1–189	24, 2–229	0.913
			
*Total follow-up from diagnosis of first DT to last observation (years)*			
Median, min–max	8, 0–29	7, 0–28	0.762
			
*Age at last follow-up (years)*			
Median, min–max	41, 23–67	42.5, 18–79	0.606
			
*Status at last follow-up, N* (%)			
Alive	21 (58)	18 (69)	0.783
Lost to follow-up	1 (3)	1 (4)	
Dead due to DT	9 (25)	5 (19)	
Dead due to other cause	5 (14)	2 (8)	
			
*DT status at last follow-up, N* (%)			
Regression/stable	25 (69)	20 (77)	0.515
Progression/variable	11 (31)	6 (23)	

Abbreviations: DT=desmoid tumour; FAP=familial adenomatous polyposis.

**Table 2:** Characteristics and follow-up data of extra-abdominal and abdominal wall desmoid tumours

	**Extra-abdominal and abdominal wall DT (*N*=16)**
*Sex, male, N* (%)	5 (31)
*DT localisation, N* (%)	
Abdominal wall	13 (81)
Trunk	2 (13)
Head/neck	1 (6)
	
*Age at first DT (years)*	
Median, min–max	30.5, 8–57
	
*Size of first DT (cm)*	
Median, min–max	5, 2–13
	
*Primary treatment, N* (%)	
Surgery	13 (81)
Surgery and medication	1 (6)
Medication	1 (6)
Wait-and-see	1 (6)
	
*DT progression, N* (%)	5 (31)
	
*Time to DT growth months*	
Median, min–max	30 (2–73)
	
*Status last follow-up, N* (%)	
Alive	13 (81)
Dead due to DT	0
Dead due to other cause	3 (19)
	
*Desmoid status at last follow-up, N* (%)	
Regression/stable	12 (75)
Progression/variable	4 (25)

Abbreviation: DT=desmoid tumour.

**Table 3:** Description of treatment outcome of patients who received cytotoxic chemotherapy and/or targeted agents as desmoid treatment

**Sex**	**Site DT**	**Age (years)**	**Treatment**	**Effect on desmoid growth**	**Follow-up (months)**
Male	Mesentery	45	Irresectable DT, etoposide and ifosfamide, tamoxifen tamoxifen and LHRH-agonist anastrozole	Quick regression DT, necrosis in DT Stabilisation, after 5 years progression Progression	5 70 5
Male	Head, abd. wall and mesentery	15–17	R_2_ resection DT head, RT mes. DT, sulindac, toremifene doxorubicine and DTIC, R_2_ resection mes. DT sulindac, toremifene, R_2_ resection abd. wall DT all medication stopped	Progression mes. DT Stabilisation, after 2 years abd. wall DT Both periods of progression and regression, after 2 years growth DT head, DT mes. and abd. wall stable Stabilisation	35 38 100 36
Male	Mesentery	29	R_2_ resection, sulindac, toremifene doxorubicine and carboplatin R_2_ resection, sulindac, tamoxifen	Progression Regression <25% Stabilisation	11 7 50
Male	Mesentery	29	R_2_ resection, sulindac, tamoxifen, toremifene doxorubicine and ifosfamide, sulindac, toremifene imatinib	Progression Stabilisation, after 8 months progression Stabilisation, but fistulas and abscesses at DT	38 8 10
Female	Abd. wall, trunk, breasts, neck	25–40	Multiple R_2_ resections, tamoxifen, sulindac, LHRH-agonists, anastrozole, radiotherapy imatinib	Progression and multiple new DT Progression Progression	11 10
Male	Mesentery	32	R_2_ resection doxorubicine and DTIC	Progression	19
				Regression, death not due to DT	184
Male	Mesentery	30	Chemotherapy^a^ and radiotherapy, R_2_ resection colchicine, LHRH-agonists, anti-estrogens, prednison, IFN	Stabilisation for 4 years Progression; after colchicine multiple abscesses; death due to DT	51 58
Female	Mesentery and abd. wall	24	Naproxen, toremifene doxorubicine and DTIC	Progression Progression, death due to DT	16 3
Female	Mesentery	33–35	Sulindac, anti-estrogens, DT irresectable liposomal doxorubicine	Progression Death pulmonary embolism, due to compression of DT on the large veins	24 0
Female	Mesentery	35–37	Wait-and-see, sulindac, celecoxib, tamoxifen, toremifene carboplatin and doxorubicine imatinib fulvestrant	Progression Necrosis in DT, fistulas and abscesses Stabilisation, after 1 year progression Stabilisation, after 2 years progression and death due to DT	37 7 12 19
Male	Mesentery	47	Irresectable DT, sulindac, tamoxifen vinblastin and methotrexat	Progression Progression, death due to desmoid	4 18

Abbreviations: abd. wall=abdominal wall; DT=desmoid tumour; DTIC, dacarbazine; IFN=interferon; LHRH, luteinizing hormone releasing hormone; Mes.=mesenterial; RT=radiotherapy.

a Details and type of chemotherapy are not available.
